# The Role of Spirituality and Religiosity in Subjective Well-Being of Individuals With Different Religious Status

**DOI:** 10.3389/fpsyg.2019.01525

**Published:** 2019-07-09

**Authors:** Daniela Villani, Angela Sorgente, Paola Iannello, Alessandro Antonietti

**Affiliations:** Department of Psychology, Università Cattolica del Sacro Cuore, Milan, Italy

**Keywords:** subjective well-being, spirituality, religiosity, religious status, life satisfaction, positive affect, negative affect, religious identity

## Abstract

Spirituality and religiosity have been found to be positive predictors of subjective well-being, even if results are not altogether consistent across studies. This mixed evidence is probably due to the inadequate operationalization of the constructs as well as the neglect of the moderation effect that the individuals’ religious status can have on the relation between spirituality/religiosity and subjective well-being. The current study aimed to investigate the relationship of spirituality and religiosity with subjective well-being (operationalized as both life satisfaction and balance between positive and negative affect) and to test whether differences exist according to individuals’ religious status (religious, non-religious, and uncertain). Data were collected from 267 Italian adults aged 18–77 (*M* = 36.68; SD = 15.13), mainly women (59.9%). In order to test the role of spirituality (operationalized as Purpose, Innerness, Interconnection, and Transcendence) and religiosity (operationalized as three dimensions of the religious identity: Commitment, In-depth Exploration, and Reconsideration of Commitment) in subjective well-being, two path analysis models were run, one for each predictor. To test the invariance of the two models across the individuals’ religious status, two multi-group models were run. The models concerning spirituality were tested on the entire sample, finding that spirituality had a positive impact on subjective well-being (except for the dimension of Interconnection) and that this relation is unaffected by the individual’s religious status. The models concerning religiosity were instead tested only on religious and uncertain, finding that the relationship between religiosity and subjective well-being changes across religious status. In particular, the main difference we found was that religious identity commitment positively predicted satisfaction with life among religious, but not among uncertain individuals. An interpretation of the results and their implications are discussed.

## Introduction

Subjective well-being (SWB) concerns people’s evaluations of the quality of their own lives (Diener, 1984; Stratham and Chase, 2010). This appraisal comprises a cognitive and an affective component (Diener, 1984; Luhmann, 2017; Diener et al., 2018), which refer, respectively, to cognitive judgments about achieving important values and goals in the life span of the individual and to the balance between positive and negative affect (Luhmann et al., 2012a,b). Thus, SWB corresponds to an overall satisfaction with one’s life (e.g., Diener, 1984) and long-term levels of happiness that result from a global self-evaluation of whether individuals are living a good existence or not (Diener and Seligman, 2004; Diener et al., 2009a; Diener, 2012).

In the literature, the affective dimension of SWB has been alternatively operationalized and measured as the presence of positive well-being (e.g., happiness; Sagiv and Schwartz, 2000; Pollard and Lee, 2003), the prevalence of positive affect [e.g., the Positive and Negative Affect Schedule (PANAS), Watson et al., 1988; the Scale of Positive and Negative Experience (SPANE), Diener et al., 2009b], or the absence of negative affect (Cummins, 2013). The cognitive dimension of SWB – that is life satisfaction – has been measured through both the Satisfaction With Life Scale (SWLS; Diener et al., 1985), which refers to a global evaluation of life satisfaction (e.g., Mak et al., 2011), and the Personal Well-being Index (PWI; Cummins and Lau, 2004), which requires a domain evaluation of life satisfaction (Lai et al., 2013).

Different aspects may contribute to and influence how people appraise the many facets of their lives, ranging from individual characteristics that distinguish between happy and unhappy personalities, to values people consider important and worth pursuing in life or the fulfillment of social needs (Balzarotti et al., 2016; Diener et al., 2018; Schwartz and Sortheix, 2018). Among others, a growing body of research investigates the role that spirituality and religiosity play in individuals’ self-perceived well-being, identifying a positive effect of religion and spirituality on many psychosocial and health-related outcomes across the lifespan (e.g., Fabricatore et al., 2000; Fry, 2000; Mueller et al., 2001; George et al., 2002; Levin and Chatters, 2008; Krause, 2011; VanderWeele, 2017).

Given the complexity of religiosity and spirituality constructs, it turns out to be critical to specify how these concepts have been conceptualized in literature. In line with Pargament (1997), religiosity and spirituality are intended in terms of the individual’s values, beliefs, behaviors, and identity, which may focus on either the sacred or the functional aspects of religion.

Specifically, on the one hand, religiosity is often seen as “the formal, institutional, and outward expression” (Cotton et al., 2006, p. 472) of one’s relationship with the sacred, and it is typically operationalized as beliefs and practices associated with a particular religious worldview and community (Iannello et al., 2019). On the other hand, spirituality is conceptualized as the search for meaning in life, for a personal connection with transcendent realities, and for interconnectedness with humanity (Zinnbauer et al., 1999; Benson and Roehlkepartain, 2008; Worthington et al., 2011), and it is thus operationalized as the human desire for transcendence, introspection, interconnectedness, and the quest for meaning in life (King and Boyatzis, 2015), which can be experienced in and/or outside of a specific religious context (Benson et al., 2003).

### Association Between Religiosity, Spirituality, and Subjective Well-Being

Spirituality and religiosity have been found to be positive predictors of SWB, even if results are not altogether consistent across studies (Kim-Prieto and Miller, 2018). Concerning the cognitive dimension of SWB, a number of studies found a positive relationship between spirituality as well as religiosity and life satisfaction (Yoon and Lee, 2004). To explain these findings, it has been suggested that people who experience more connection with and direction from a higher power, that is, people who show high religious and spiritual involvement, tend to give a more positive appraisal of their lives (Vishkin et al., 2016, 2019; Ramsay et al., 2019). The sense of being in connection with a higher power, with others, and, in general, with life represents an effective way to maintain a positive evaluation of one’s life, despite all the possible negative circumstances that one may encounter. Additionally, religious and spiritual involvement may benefit individuals’ lives through empowering both internal (e.g., feeling of self-worth) and social (e.g., sense of belonging to a network) resources (Lim and Putnam, 2010).

Further support to this view consists in the role of religious beliefs and practices that are usually positively related to life satisfaction (Koenig and Larson, 2001; Abu-Raiya et al., 2015; Krause, 2015). Holding beliefs with strong conviction, whether referring to the existence or non-existence of God, may itself exert a salutary effect and enhance individual well-being by reducing the cognitive dissonance. In the absence of subjective certainty, people could experience a state of psychological tension that they are motivated to reduce (Kahneman et al., 1982; Kitchens and Phillips, 2018). This could be the underlying reasons to the fact that once religious and non-religious individuals are fairly compared regarding the strength of their beliefs, they report a similar level of well-being, as showed by Galen and Kloet (2011).

To better understand the role of religiosity on SWB, it is also important to consider how religiosity is conceived within the specific background culture. For example, Graham and Crown (2014) used a large-scale dataset including about 160 nations, and they found an overall positive relation between religiosity and SWB moderated by culture. Specifically, in cultures with high levels of religiosity, being religious had a greater impact on SWB, compared to cultures with low levels of religiosity. The same result has been found by Stavrova et al. (2013): by using the European and World Values Studies datasets, the authors found that the predictive power of religiosity on life satisfaction was greater in highly religious cultures, whereas the relation was negative in cultures that valued atheism.

However, other research failed to find any connection between religiosity and life satisfaction (Kirkpatrick and Shaver, 1992; Mak et al., 2011), thus questioning the existence of a direct relationship between individuals’ beliefs as well as attitudes toward religion and their own satisfaction with life.

As for the effect of religiosity and spirituality on the affective dimension of SWB, findings are mixed as well. Some studies, which reported a weak relationship between religiosity/spirituality and positive affect (Diener et al., 2011; Lun and Bond, 2013), highlighted a possible effect of the social structure provided by religious affiliation on experiencing positive affect.

In particular, it seems that some practices – such as prayer – positively contribute in inducing positive states such as gratitude (Lambert et al., 2009). Moreover, recent studies report the role played by self-transcendent emotions, such as awe, hope, love, and forgiveness in mediating the relationship between religion and well-being (Van Cappellen et al., 2016). These studies emphasize the role of religiosity in the induction of positive emotions (Fredrickson, 2002).

Furthermore, according to Ramsay et al. (2019), another important mechanism that can explain the relationship between religiosity and well-being is that of emotional regulation, which consists in the modulation of emotional states functionally to the environment’s demands. To the extent that religion constantly trains people to reassess emotional events, religious individuals may become more used to cognitive reappraisal. These hypotheses have recently been confirmed by studies by Vishkin et al. (2016), even among individuals of different religions (Vishkin et al., 2019).

Other studies failed to report a correlation between religiosity/spirituality and positive/negative affect (Fabricatore et al., 2000), thus suggesting that being more religiously involved and spiritually integrated does not relate significantly to one’s affective experience.

A possible explanation of the inconsistency of findings across studies might lie in the different operationalization of these constructs and in the diverse instruments used to measure them. Both religiosity and spirituality have been defined and measured differently across studies. Multiple and different indicators of religiosity and spirituality have been associated with SWB, thus accounting, at least partly, for the mixed evidence (Lun and Bond, 2013).

### The Present Study

The literature about the relationship between religiosity, spirituality, and SWB has not yet achieved consistent results (Lun and Bond, 2013; Kim-Prieto and Miller, 2018), and we argue that there are three main general flaws in this research field.

First, the theoretical framework used to define and measure SWB as associated with religiosity and spirituality has often been too broad and focused only on the cognitive or the affective dimension of SWB, thus leading to an incomplete investigation (Lim and Putnam, 2010). To overcome this weakness, in the present study, we clarified the theoretical reference model about SWB as including both a cognitive and an affective component (Diener, 1984; Luhmann, 2017; Diener et al., 2018), and we used the typical measures to assess them, which are life satisfaction and balance between positive and negative affect (Diener et al., 1985; Watson et al., 1988; Luhmann et al., 2012a,b).

Second, religiosity and spirituality constructs appear in literature as distinct even if interconnected (Zinnbauer et al., 1999; Hill and Pargament, 2008), and the studies have typically considered only one of the two and its association with SWB (Fabricatore et al., 2000; Lun and Bond, 2013; Kim-Prieto and Miller, 2018). Such a basic distinction may not be helpful for understanding how religion and spirituality differ in their associations with dimensions of SWB. In the present study, we operationalized religiosity in terms of religious identity, which refers to the extent to which people see their religious beliefs, practices, and community belonging as central to the representation that they have of themselves and that they want to give outside of themselves (Lopez et al., 2011). Spirituality instead was operationalized as the human desire for transcendence, introspection, interconnectedness and the quest for meaning in life (King and Boyatzis, 2015). The distinct role of religiosity and spirituality on SWB has been tested through two separate path analysis models.

Third, we noticed that the grouping of religious experience reported on a subjective level was not univocal (Galen and Kloet, 2011; Kitchens and Phillips, 2018). In several studies, those with weak belief (low or weakly religious) and those with complete non-belief (completely non-religious or atheists) have been conflated in one group, thus combining opposite poles on the certainty of belief dimension (i.e., weakly religious with confidently non-religious). This grouping made it difficult to compare the obtained results. Following the suggestion by Galen and Kloet (2011), in the present study, we distinguished participants according to their religious status without collapsing the completely non-religious individuals and the uncertain ones.

Specifically, starting from these premises, the present study aims at (1) investigating the role of religiosity and spirituality on the cognitive and affective dimension of SWB and (2) studying whether the relationship between religiosity/spirituality and SWB varies according to the individuals’ religious status (religious, non-religious, uncertain).

## Materials and Methods

An advertisement for research participation containing a hyperlink to a questionnaire on a secure server of the Psychology Department was sent by email to students’ and researchers’ personal contacts. Then, the sample was recruited through non-random snowball sampling.

The online survey took approximately 25 min to complete. Participating in the survey was entirely voluntary without any form of compensation. All subjects gave written informed consent in accordance with the Declaration of Helsinki. The protocol was approved by the Ethical Committee of the Department of Psychology of Università Cattolica del Sacro Cuore of Milan.

### Participants

Data were collected from December 2017 to May 2018. The convenient sample was composed of 267 Italian adults aged 18–77 (*M* = 36.68; SD = 15.13), mainly women (59.9%). For what concerns their religious status, most of the participant reported to be religious (58.1%), whereas 14.2% stated they were non-religious. The remaining 27.7% of participants were declared to have unsure beliefs about their religious status as they stated they were neither religious nor non-religious. Only to religious and uncertain participants we asked to select which religious they belong to and 95.9% of them reported to be Christian (mainly Catholic).

In order to validate the religious statuses (religious, non-religious, and uncertain) that participants attributed to themselves, we assessed behavioral indicators of religiosity (Fincham et al., 2010; Krause, 2010) by asking them to report the frequency of their attendance to religious services as well as the frequency of their praying on a 5-point scale (0 = never; 1 = only in special occasions; 2 = rarely; 3 = at least once a month; 4 = at least once a week; 5 = every day or almost every day).

Religious participants stated they attended church services at least once a month (*M* = 2.97; SD = 1.33) and to prayed at least once a week (*M* = 3.74; SD = 1.50). Non-religious participants reported that they do attend religious services (*M* = 0; SD = 0) and do not pray (*M* = 0.27; SD = 1.08). Finally, people that felt to be between religious and non-religious (i.e., uncertain) stated that they attended religious services (*M* = 1.19; SD = 1.03) and prayed (*M* = 1.10; SD = 1.31) only in special occasions.

### Measures

#### Spirituality

Spirituality was assessed using the Italian version (Iannello et al., 2019) of the 28-item Spirituality Assessment Scale (Howden, 1992). Items were rated on a 6-point scale from 1 (strongly disagree) to 6 (strongly agree) and belonged to four different subscales. Specifically, the Purpose subscale is composed of four items (sample item: “My life has meaning and purpose”), and the Innerness (sample item: “I have an inner strength”) and the Interconnection (sample item: “I have a general sense of belonging”) subscales are both composed of nine items, while the Transcendence subscale (sample item: “Even when I feel discouraged, I trust that life is good”) is composed of six items. As this scale is not yet validated on the Italian population, we verified that the expected factorial structure fitted well our data, obtaining sufficient fit indices: *χ*^2^(307) = 590.13; *p* < 0.001; RMSEA = 0.06 (0.05, 0.07); *p* = 0.010; CFI = 0.900; SRMR = 0.06. This scale resulted to be also highly reliable. The Cronbach’s α for the four subscales was α = 0.835, α = 0.846, α = 0.801, and α = 0.713, respectively.

#### Religiosity

Religious identity formation was measured by the 13-item Utrecht-Management of Identity Commitments Scale (U-MICS; Crocetti et al., 2008, 2010) that assesses three identity formation processes (commitment, in-depth exploration, and reconsideration of commitment) within the religious domain (Iannello et al., 2019). Specifically, individuals must make identity commitments, such as to particular religious worldviews, but then they can either deepen those commitments through in-depth exploration – which involves the desire to reflect, learn, and share their commitments – or step back and reconsider those commitments, perhaps in preparation to disengage from them and redirect toward different religious beliefs (Crocetti et al., 2008). The U-MICS scale has been already validated in Italy (Crocetti et al., 2010), but in domains other than religious identity. Consequently, we verified that the expected three-factor structure was confirmed also on our sample. We obtained sufficient fit indices: *χ*^2^(62) = 162.03; *p* < 0.001; RMSEA = 0.08 (0.07, 0.10); *p* < 0.001; CFI = 0.938; SRMR = 0.04.

As expected, the scale is composed of three subscales, each corresponding to a different identity formation process: the 5-item Commitment subscale (sample item: “My religion gives me security in life”), the 5-item In-depth exploration subscale (sample item: “I try to find out a lot about my religion”), and the 3-item Reconsideration of commitment subscale (sample item: “I often think that a different religion would make my life more interesting”). Items were rated on a 5-point scale from 1 (completely untrue) to 5 (completely true). All the subscales were highly reliable, respectively α = 0.936, α = 0.906, and α = 0.864. This scale was administered only to participants who reported to be religious or uncertain, as we argued that non-religious had a religious status that could not allow them to answer items referring to “my religion.”

#### Life Satisfaction

The cognitive dimension of the SWB was measured by the Italian version of the Satisfaction with Life Scale (SWLS; Diener et al., 1985; Di Fabio and Busoni, 2009). The scale is composed of five items (sample item: “If I could live my life over, I would change almost nothing.”) eventuated of a 7-point scale (1 = strongly disagree; 7 = strongly agree). Internal consistency of the scale was high (α = 0.862).

#### Positive and Negative Affect

The emotional dimension of the SWB was measured by the Italian version of the Positive and Negative Affect Schedule (PANAS; Watson et al., 1988; Terraciano et al., 2003). It consists of a list of 20 adjectives used to describe different feelings and emotions: 10 positive moods/emotions and 10 negative moods/emotions. Participants must indicate if they feel these emotions in that moment with a 5-point scale (1 = not at all; 5 = completely). Both the 10-item Positive Affect subscale (sample item: “interested”) and the 10-item Negative Affect (sample item: “nervous”) were highly reliable, respectively, α = 0.884 and α = 0.897.

### Data Analysis

First, descriptive statistics were run for all the variables involved in the current study, separately for the three groups here investigated (religious, non-religious, and uncertain). SPSS (Version 20; IBM Corp., 2011) software was adopted. Second, the relationships between predictors (spirituality and religiosity) and outcome (SWB) were tested performing path analysis models separately for each predictor.

All models were run in Mplus (version 7; Muthén and Muthén, 1998–2014). As suggested by Rhemtulla et al. (2012), variables measured on a 5- or more-point Likert scale were treated as continuous, allowing the adoption of Maximum Likelihood as estimator. Missing on each item, ranging from 0 to 7.83%, resulted in Missing Completely at Random [Little test’s *χ*^2^ (114) = 139.194; *p* = 0.054] and was managed using the Full Information Maximum Likelihood method.

#### Spirituality and Subjective Well-Being

The model testing the relationship between spirituality (measured by four subscales: Purpose, Innerness, Interconnection, and Transcendence) and SWB (measured by three dimensions: Life Satisfaction, Positive Affect, and Negative Affect) was run on the entire sample (*n* = 267), assuming that spirituality can be experienced regardless of religious status. As correlations were required among the four predictors’ dimensions as well as among the three outcomes’ dimensions, the model was saturated and it automatically fits the data perfectly.

In order to verify if the relationships found in the model run on the entire sample were invariant across the different religious statuses (religious, non-religious, and uncertain), a multi-group model was run where all the correlational and regression paths were constrained to be the same across groups. Since this alternative model was not saturated, overall fit indexes were meaningful. Model fit was evaluated adopting the following indexes: *χ*^2^ value, Root Mean Squared Error of Approximation (RMSEA), and Comparative Fit Index (CFI). The model *χ*^2^ is a measure of poor fit, such that large, significant *χ*^2^ values indicate that the model fits the data poorly, whereas non-significant *χ*^2^ values indicate that the model is consistent with the data. Additionally, RMSEA is a measure of poor fit, and values close to zero indicate better fit (i.e., values less than 0.08 indicate reasonable fit and values below 0.05 indicate good fit; Marsh et al., 2004). By contrast, CFI is a measure of goodness of fit, with values close to 1 indicating a good model. However, CFI values less than 0.90 indicate that the model does not fit the data well (Marsh et al., 2004).

A *χ*^2^ difference test (Bollen, 1989) was used to evaluate whether adding the equality constraint (i.e., imposing all the paths to be the same across different religious statuses) led to significant decrement in fit. As the baseline model (i.e., the model in which the paths were freely estimated separately for each group) was a saturated model, the constrained model’s *χ*^2^, when non-significant (*p* > 0.05), indicated that the relation between spirituality and SWB was invariant across religious, non-religious, and uncertain. Vice versa, significant *χ*^2^ (*p* < 0.05) of the constrained model indicated that at least one path was significantly different across groups. In this case, the constrained model had to be modified by setting one path “free” (non-invariant) in one of the three groups. As suggested by Dimitrov (2010), the path to start freeing was selected based on modification indices reported in Mplus output.

#### Religiosity and Subjective Well-Being

The model testing the relationship between religious identity formation (measured by three subscales: Commitment, In-depth Exploration, and Reconsideration of Commitment) and SWB (measured by three dimensions: Life Satisfaction, Positive Affect, and Negative Affect) was run on a sub-sample (*n* = 229), composed only of religious and uncertain, as we did not administer items about religiosity to those who were non-religious. As correlations were required among the three predictors’ dimensions as well as among the three outcomes’ dimensions, this model was saturated.

In order to verify if the relationships found in this model were invariant between religious and uncertain, a multi-group model was run where all the correlational and regression paths were constrained to be the same across the two groups.

As for the spirituality models, model fit was evaluated by *χ*^2^ value, RMSEA, and CFI (Marsh et al., 2004). A *χ*^2^ difference test (Bollen, 1989) was used to compare the free and the constrained models. If full invariance was not reached (i.e., significant *χ*^2^), one path at a time was freeing according to modification indices (Dimitrov, 2010).

## Results

### Descriptive Statistics

In Table 1, we reported the mean and the standard deviation for each variable investigated in this study, separately for diverse participants’ religious statuses (non-religious, uncertain, and religious). Statistics about religiousness dimensions are not available for non-religious participants as the instrument measuring religious identity was not administered to them.

**Table 1:** Descriptive statistics separately for the participants’ religious status.

		Non-religious (*n* = 38)	Uncertain (*n* = 74)	Religious (*n* = 155)
		*M* (SD)	*M* (SD)	*M* (SD)
Religiousness	Commitment	NA	2.14 (0.84)	3.32 (0.77)
	In-depth exploration	NA	2.45 (0.90)	3.38 (0.81)
	Reconsideration of commitment	NA	1.95 (0.80)	1.49 (0.68)
Spirituality	Purpose	4.04 (1.16)	4.36 (0.92)	4.61 (0.86)
	Innerness	3.53 (0.89)	3.83 (0.81)	4.28 (0.79)
	Interconnection	4.07 (0.77)	4.18 (0.77)	4.50 (0.65)
	Transcendence	3.50 (0.94)	3.73 (0.84)	3.94 (0.76)
Subjective well-being	Life satisfaction	4.30 (1.32)	4.53 (1.00)	4.67 (1.15)
	Positive affect	2.80 (0.98)	2.89 (0.80)	3.12 (0.75)
	Negative affect	1.71 (0.83)	1.83 (0.71)	1.79 (0.78)

*Note: NA, not applicable*.

### Spirituality and Subjective Well-Being

**Figure 1:**
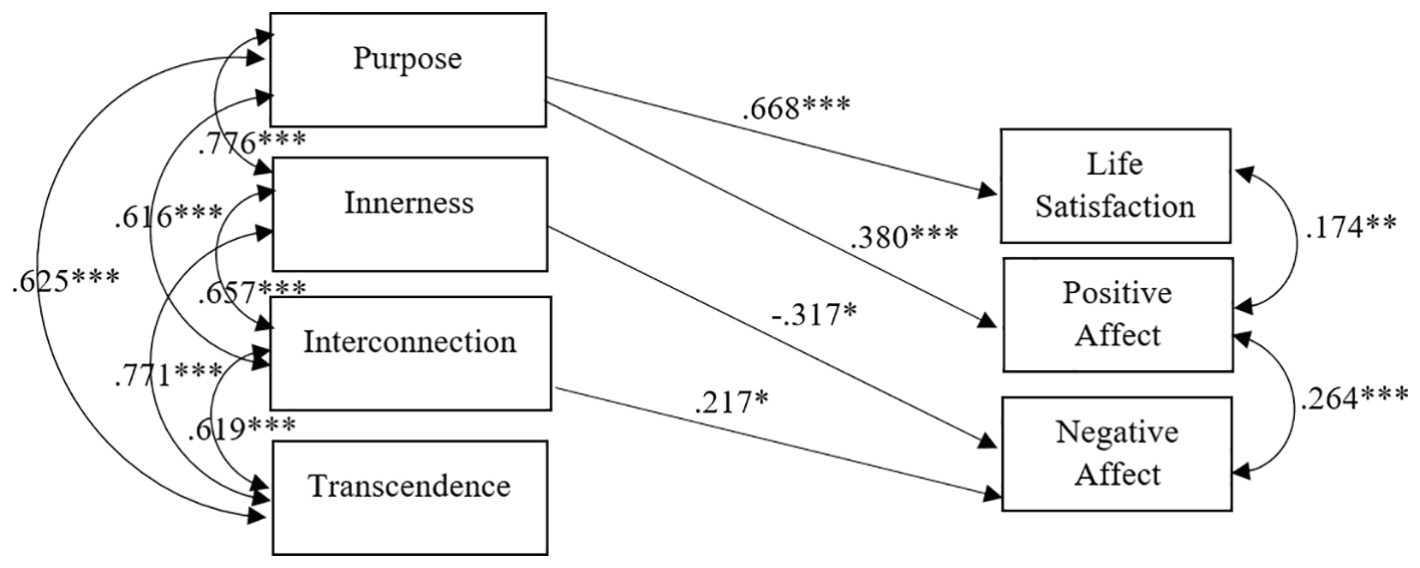
Path analysis testing the influence of spirituality on SWB (*n* = 267). Only significant correlational and regression paths are represented (^*^p < 0.05; ^**^p < 0.01; ^***^p < 0.001). Standardized values are reported.

Results of the saturated model testing the relationship between spirituality (measured by four subscales: Purpose, Innerness, Interconnection, and Transcendence) and SWB (measured by three dimensions: Life Satisfaction, Positive Affect, and Negative Affect) were reported in Figure 1. This model was run on all the participants (religious, non-religious, and uncertain).

In order to verify if the model represented in Figure 1 works equally for religious, non-religious, and uncertain, a multi-group model was run where all the (significant and non-significant) correlational and regression paths were constrained to be the same across groups. This constrained model had very good fit indices [*χ*^2^(42) = 44.62; *p* = 0.36; RMSEA = 0.026 (0.000, 0.078); *p* = 0.71; CFI = 0.989]. Furthermore, the non-significant *χ*^2^ showed that the impact of the spirituality on the SWB is the same regardless of the individual’s religious status.

### Religiosity and Subjective Well-Being

**Figure 2:**
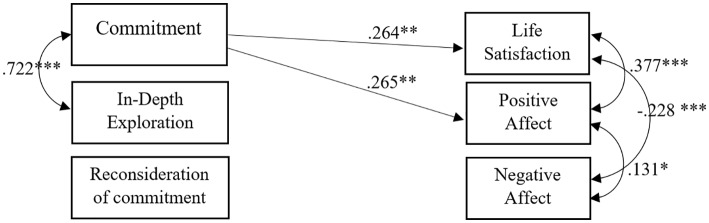
Path analysis testing the influence of religious identity formation on SWB (*n* = 229). Only significant correlational and regression paths are represented (^*^p < 0.05; ^**^p < 0.01; ^***^p < 0.001). Standardized values are reported.

Results of the saturated model testing the relationship between religious identity formation (measured by three subscales: Commitment, In-depth Exploration, and Reconsideration of Commitment) and SWB (measured by three dimensions: Life Satisfaction, Positive Affect, and Negative Affect) were reported in Figure 2. This model was run only on religious and uncertain.

In order to verify if the relationships reported in Figure 2 were invariant between religious and uncertain, a multi-group model was run where all the (significant and non-significant) correlational and regression paths were constrained to be the same across the two groups. The model fully constrained resulted to be non-invariant between religious and uncertain (significant *χ*^2^; see Table 2). In order to reach a constrained model non-significantly different (i.e., non-significant *χ*^2^) from the baseline model (i.e., model with parameters free to be different between religious and uncertain), four parameters were successively made free (see Table 2). Four parameters non-invariant between the two religious statuses were reported in Table 3.

**Table 2:** Fit indices of models testing the relationship between religiosity and subjective well-being.

	*χ*^2^	df	*p*	RMSEA (90% CI)	*p*	CFI
Full constrained model	32.373	15	0.006	0.101 (0.052, 0.148)	0.044	0.808
- Less life satisfaction on commitment	30.109	14	0.007	0.100 (0.050, 0.150)	0.050	0.822
- Less negative affect on commitment	25.716	13	0.019	0.092(0.037, 0.145)	0.091	0.859
- Less life satisfaction on in-depth exploration	22.114	12	0.036	0.086(0.021, 0.141)	0.138	0.888
- Less reconsideration of commitment with in-depth exploration	18.575	11	0.069	0.078(0.000, 0.137)	0.207	0.916

**Table 3:** Religiosity model’s non-invariant parameters between religious and uncertain.

	Religious (*n* = 155)	Uncertain (*n* = 74)
Life satisfaction on commitment	*β* = 0.565; *p* < 0.001	*β* = −0.006; *p* = 0.973
Negative affect on commitment	*β* = −0.110; *p* = 0.262	*β* = 0.177; *p* = 0.112
Life satisfaction on in-depth exploration	*β* = −0.115; *p* = 0.356	*β* = 0.243; *p* = 0.122
Reconsideration of commitment with in-depth exploration	*r* = 0.073; *p* = 0.113	*r* = 0.227; *p* = 0.003

*Note: Non-standardized values are reported as they made comparisons more interpretable*.

**Figure 3:**
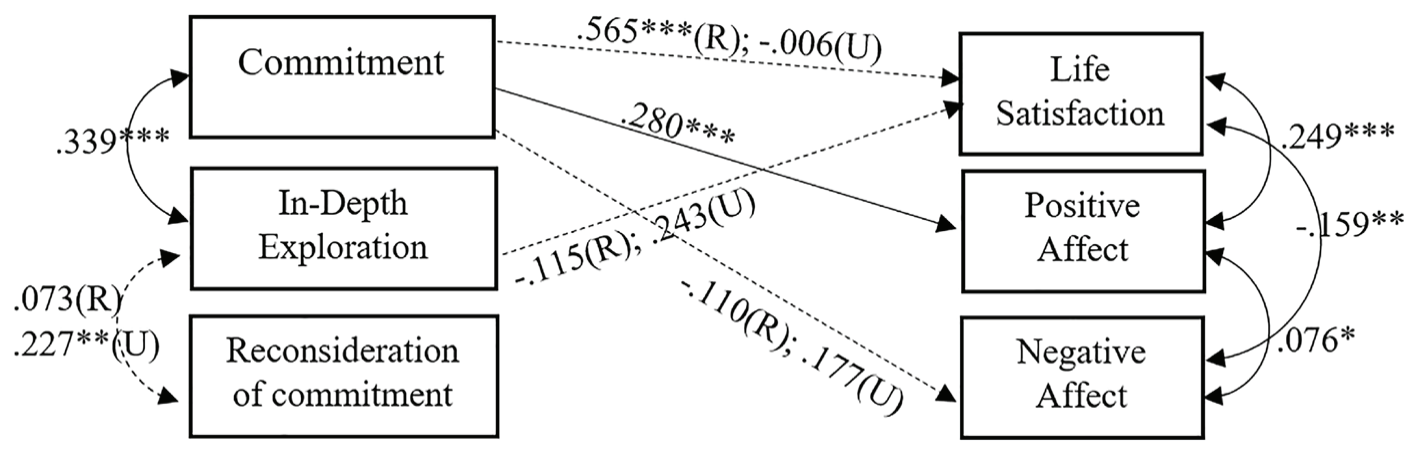
Partial invariant model between religious (R) and uncertain (U) testing the influence of religious identity formation on SWB. Only significant and/or non-invariant (i.e., dotted line) correlational and regression paths are represented (^*^p < 0.05; ^**^p < 0.01; ^***^p < 0.001). Non-standardized values are reported as they made comparisons across groups more interpretable.

In summary, the impact of spirituality on SWB can be considered invariant regardless of the individual’s religious status. In other words, what is reported in Figure 1 works for religious, uncertain, and non-religious. Instead, the impact of religiosity on SWB differs according to the individual’s religious status. Specifically, in Figure 3, we show in the solid line what is valid regardless of the religious status and, in the dotted line, what works differently for religious (R) and uncertain (U).

## Discussion

This study aimed to investigate the role of spirituality and religiosity on SWB and to test whether differences exist according to individuals’ religious status (i.e., religious, non-religious, and uncertain). By looking at the different aspects of religion and spirituality in terms of their connection to the dimensions of SWB, the present analysis yielded interesting patterns of results.

### Spirituality and Subjective Well-Being

Concerning the relationship between spirituality and SWB, we found a strong impact of spirituality – intended as the human desire for transcendence, introspection, interconnectedness, and the quest for meaning in life (King and Boyatzis, 2015) – on SWB, and this relationship appears the same regardless of the individual’s religious status. Specifically, the spirituality dimension that was strongly connected with SWB, both in its cognitive and affective aspects, was that of purpose and meaning in life. According to Speed et al. (2018), the drive to construct meaning or purpose in life is a quintessential consequence of being human rather than something that is conceived under a specific religious or philosophical framework. Thus, our results appeared as coherent with other studies that already showed the association between meaning in life and SWB (Fabricatore et al., 2000; Steger et al., 2009). Furthermore, purpose in life, which addresses the extent to which individuals perceive their lives as having goals and meaning, has already been associated with positive affect (Chen et al., 2019).

Innerness – intended as the perception of inner peace and inner strength in time of difficulties – was being negatively related to negative affect. In other words, we found that perceiving to have inner strength reduce the experience of negative affect. To understand this result, we could hint at the construct of self-efficacy and defined as the individual’s confidence in producing designated levels of performance and achieving what he/she wants (Bandura, 1997). Several studies have found that people high in self-efficacy experience higher SWB than people with low self-efficacy (Caprara and Steca, 2005; Lent et al., 2005; Strobel et al., 2011). Furthermore, Lightsey et al. (2006) found that generalized self-efficacy may play a role in the development of self-esteem, conceived as the general assessment of one’s overall self-worth, which may help in shaping negative affect.

Surprisingly, we found that interconnection – intended as a sense of belonging and connectedness to others and to the environment – was positively related to negative affect. Whereas some studies have shown the possibility of negative interaction within religious groups and congregations and the deleterious impact of this interaction on well-being (Krause et al., 2000), the negative effect from a spiritual point of view has been less investigated. It is, however, plausible to think that sharing experiences within other individuals, regardless of their belonging to faith or religious groups, may imply possible relational difficulties and negative emotional experiences. Future research is encouraged to deepen this relationship.

Contrary to our expectations, transcendence was not associated with SWB. We expected to find a positive association between the transcendence dimension and the affective dimension of the SWB, as already suggested by Van Cappellen and Rimé (2013). Indeed, the authors proposed that positive emotions and Self-Transcendence are intertwined; positive emotional states create an opened and broadened mindset favorable to self-transcendence.

However, in a content review of several notable spirituality measures, including the Spirituality Assessment Scale (Howden, 1992), de Jager Meezenbroek et al. (2012) stated that the formulation of several items of that Scale is inappropriate. Items of the transcendence scale, such as “I have the ability to rise above or go beyond a physical or psychological condition” and “The boundaries of my universe extend beyond usual ideas of what space and time are thought to be,” do not require people to reflect about firsthand experience and probably have an inconsistent meaning because of the figurative language and abstract concepts. This lack of clarity in items formulation probably did not allow us to clearly test the link between transcendence and SWB.

### Religiosity and Subjective Well-Being

Concerning the relationship between religiosity and SWB, we found that having a commitment towards a particular religion worldview helps both religious and uncertain to feel positive emotions. This result appears in line with several studies showing the role of religiosity in the induction of positive emotions (Fredrickson, 2002) and reporting that religious individuals learn more adaptive strategies to regulate their emotions (Vishkin et al., 2016). Furthermore, positive emotions have been demonstrated to be a direct consequence of behaviors related to religious commitment, such as religious attendance (Lavrič and Flere, 2008).

At the same time, having this commitment does not increase the life satisfaction in both groups. In particular, we found that religious identity commitment has a positive impact on satisfaction with life, but only in religious and not in uncertain individuals. As shown in the literature (Galen and Kloet, 2011), religious belief may assist in increasing an ideological confidence in a coherent worldview, while doubting one’s worldview is frequently associated with higher distress (Krause, 2015). This could explain why the religious commitment differently impacts well-being for religious and uncertain. Specifically, we can suppose that having a religious commitment for religious individuals increases the coherence of their life, increasing in turn the evaluation of the life satisfaction. The coherence they see in their life helps them to be satisfied with their life. Instead, this life satisfaction increase does not happen for uncertain individuals as, for them, having a religious commitment is not fully coherent with their view of life.

Results did not show an impact of in-depth exploration – intended as the process of deeply exploring one’s own religious beliefs and practices and what they mean to individuals – on SWB. Even if the same result is confirmed as not significant for religious and uncertain individuals, we noticed different coefficients across the two groups. In particular, whereas the process of in-depth exploration was positively – even not significantly – related to life satisfaction among uncertain individuals, the same process was negatively – even not significantly – associated with life satisfaction among religious individuals. Probably, in the process of inner-exploring their own religious beliefs and practices, uncertain individuals might become more open to and accepting of alternative worldviews (Saroglou, 2013), and this is associated with life satisfaction. On the contrary, for religious individuals, this kind of exploration is perceived as a threat to their own religious beliefs and, hence, negatively affects the cognitive representation of their own well-being.

Finally, the third process of the religious identity model (Crocetti et al., 2010; Iannello et al., 2019), reconsideration of commitment, referring to the efforts one makes to change no longer satisfactory present commitments, was not expected to have an impact on SWB (Karaś et al., 2015), and results confirmed our prediction.

## Conclusions and Implications

In summary, we found that both life satisfaction and affect, the two dimensions of the SWB, showed somewhat different relational patterns with measures used to assess religiosity and spirituality. As revealed by the analyses, life satisfaction, a measure of one’s cognitive well-being, was more consistently associated with both religiosity and spirituality dimensions, while affect, a measure of one’s affective well-being, appeared to be more predicted by the spirituality dimensions (if we consider the number of significant relations).

On the one hand, religiosity and spirituality are meaning-making systems and serve as ways to understand the self and the interaction with the world (Park, 2005), and they may engender perceived control and positive expectations about the future (Jackson and Bergeman, 2011; Speed et al., 2018; Chen et al., 2019). On the other hand, there is a growing literature on emotional benefits of spiritual practices. Research has shown that specific meditation practices increase positive emotions, which in turn yield positive consequences for life satisfaction (Fredrickson et al., 2008; Kok et al., 2013).

To better investigate differences in the role of religiosity and spirituality on SWB, we have to consider that other moderating variables, such as personal values one attaches to religion and spirituality, which concerns the respect, concern, and acceptance of the customs and ideas that traditional culture or religion provide the self, and other socio-cultural, cognitive, and individual variables may be important moderators of the influences on SWB (Sagiv and Schwartz, 2000; Hayward and Krause, 2014; Van Cappellen et al., 2016).

For example, Diener et al. (2011) found that the positive relationship between religiosity and SWB was mediated by social support, feelings of respect, and meaning in life. These, in turn, were moderated by difficult life circumstances. Thus, results showed that when life circumstances were difficult, greater religiosity predicted greater SWB *via* greater social support and meaning in life.

Although interesting, these findings should be considered in light of several limitations. First, due to the correlational nature of the data, caution is required in the interpretation of the relationships among the variables as observed in the current research. In our models, we assumed that religiosity and spirituality led to greater SWB. However, future longitudinal designs are necessary to better ascertain temporal ordering and causality. The relatively small sample size – in particular if considering the wide age range among participants – represents a limitation of the present study. Findings should be replicated with a larger sample, possibly focusing on specific age cohorts to explore the pattern of relationships between spirituality, religiosity, and subjective well-being in specific life stages. The third limitation is related to the need to generalize results to the national cultural context in which the relationship is examined (Lun and Bond, 2013). Thus, as the sample was mostly composed of Italian Catholic individuals, we have to be cautious in generalizing these results to other cultural contexts. Different religious orientations involve ideologies or social practices that could associate differentially with people’s SWB. Up to now, convincing and legitimate cross-religious studies have not yet been conducted (Rizvi and Hossain, 2017), and future works are encouraged to take a religion-specific perspective and to consider how religiosity is conceived within the specific background culture (Stavrova et al., 2013; Graham and Crown, 2014) to examine the relationship of religion and spirituality with well-being.

To conclude, we could say that in light of the value and the influence that spirituality and religiosity have on individuals’ subjective well-being, mental health professionals need to recognize this issue and integrate them in their work. Results coming from this study emphasize the importance of orienting clients in identifying their purpose and goals in life and this is in line with what the Self-Determination approach suggests (Ryan and Deci, 2000). Furthermore, even if we do not want to deny the importance that intrinsic orientation to religious faith has for well-being, the results of the present study lead us to not underestimate the positive impact that adherence to faith and religious practices also exerts on SWB. Thus, psychologists working in both clinical and non-clinical settings must have open conversations with their clients to be aware of the role that spirituality and religiosity may play as a stressor or a resource and develop a mutually satisfactory relationship (Shafranske and Cummings, 2013).

## Data Availability

The datasets generated for this study are available on request to the corresponding author.

## Ethics Statement

All subjects gave written informed consent in accordance with the Declaration of Helsinki. The protocol was approved by the Ethical Committee of the Department of Psychology of Università Cattolica del Sacro Cuore of Milan.

## Author Contributions

DV developed the study concept and collected data. DV, AS, and PI performed the data analysis and interpretation and wrote the first draft of the manuscript. All authors were involved in the critical revision of the manuscript and approved the final version of the manuscript.

## Conflict of Interest Statement

The authors declare that the research was conducted in the absence of any commercial or financial relationships that could be construed as a potential conflict of interest.
